# Unraveling the dual nature of brain CD8^+^ T cells in Alzheimer’s disease

**DOI:** 10.1186/s13024-024-00706-y

**Published:** 2024-02-14

**Authors:** Dan Hu, Howard L. Weiner

**Affiliations:** Ann Romney Center for Neurologic Diseases, Brigham and Women’s Hospital, Harvard Medical School, 02115 Boston, MA USA

**Keywords:** Alzheimer’s disease, CD8^+^ T cells, Microglia

CD8^+^ T cells are essential components of adaptive immunity, and primarily function as cytotoxic T lymphocytes (CTLs) that recognize and eliminate infected or abnormal cells in the body. However, a small subpopulation of CD8^+^ T cells act as regulatory T cells (CD8^+^ Tregs) that suppress immune responses [1]. For a considerable period, it was widely held that the central nervous system (CNS) was immune privileged and impervious to T cells. However, over the past decade, research conducted in both murine and human subjects has unequivocally demonstrated the existence of brain-resident memory T cells [2]. T-cell migration to specific locations in response to inflammation and infections is orchestrated by interactions between chemokines and their receptors [3]. Beta-amyloid (Aβ) plaque deposition in the brain is one of the hallmark pathologies of Alzheimer’s disease (AD). Microglia, the brain’s innate immune cells, clear Aβ plaques. As AD advances, microglia may gradually lose their ability to eliminate these plaques effectively, and, in turn, begin to generate inflammatory mediators that could potentially expedite the progression of Aβ plaque accumulation [4]. In a recent study, Su et al. established a link between chemokine-chemokine receptor interaction, brain-infiltrating CD8^+^ T cells, and microglia in AD pathogenesis (Fig. 1A). Researchers demonstrated in an Aβ-driven AD mouse model (5xFAD mice) that chemokine CXCL16, secreted by microglia, attracted peripheral blood CD8^+^ T cells expressing CXCR6, which is a receptor for CXCL16. This attraction led these CD8^+^ T cells to enter the brain and migrate to the proximity of amyloid beta (Aβ) plaques, where CXCL16-secreting microglia were also found [5]. Su et al. reported that, instead of functioning as CTLs, these CXCR6^+^CD8^+^ T cells underwent clonal expansion in the brain, becoming PD-1^+^ and operating as Tregs. They alleviated the inflammatory state of microglia, ultimately leading to a reduction in Aβ plaque burden and mitigation of cognitive decline [5]. One of the most groundbreaking aspects of Su et al.’s study lies in its observation of the protective role of CD8^+^ T cells in AD development. This is particularly noteworthy when considering another recent and highly regarded study conducted by Chen et al. in a tauopathy mouse model of AD (TE4 mice) [6]. Chen et al. also reported microglia-mediated infiltration of T cells in the brain during neurodegeneration and tauopathy-associated T cell clonal expansion. However, in this AD model, CD8^+^ T cells were identified as contributors to a detrimental role in the neurodegeneration [6] (Fig. 1B).

**Fig. 1:**
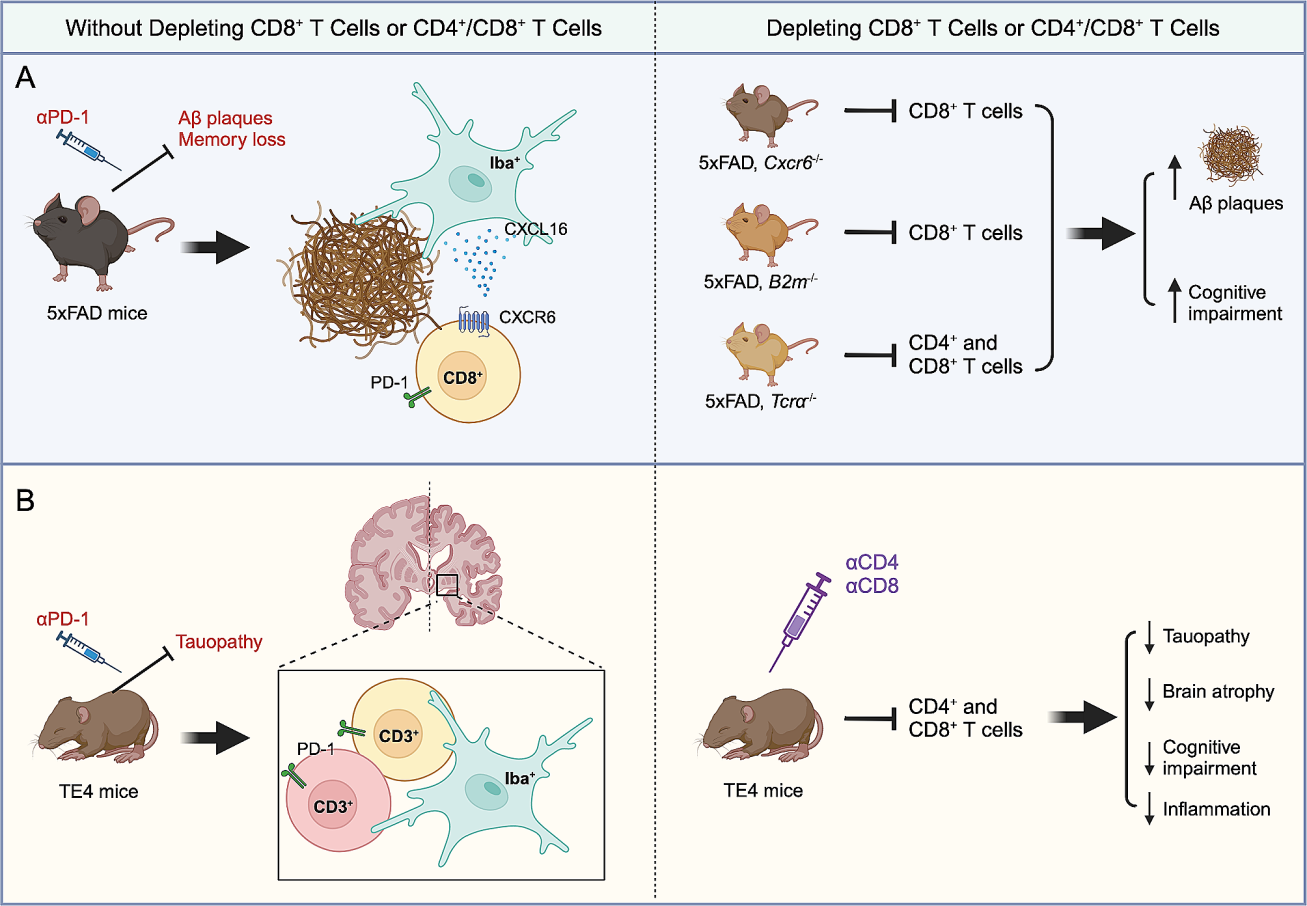
Two distinct Alzheimer’s disease mouse models for studying T cells in AD pathogenesis. (**A**) In aged 5xFAD mice with amyloid beta (Aβ) plaque deposition and cognitive impairment, an aberrantly higher presence of brain CD8^+^ T cells, but not CD4^+^ T cells or γδT cells, is linked to these AD pathologies. Among brain cells, microglia produce the highest levels of CXCL16. The communication between microglia and CXCR6-expressing CD8^+^ T cells, facilitated by CXCL16-CXCR6 interaction, allows CXCR6^+^ CD8^+^ T cells from peripheral blood to enter the brain and migrate towards Aβ plaques, where microglia are also concentrated (left panel). In the 5xFAD mice, depleting brain CD8^+^ T cells or disrupting CD8^+^ T function by creating CXCR6, B2m, or T-cell receptor alpha chain (TCRα)-deficient mice results in an increased Aβ plaque deposition and worsened cognitive impairment (right panel). These findings suggest that brain CD8^+^ T cells serve a protective role during AD development. (**B**) In aged human ApoE4-expressing P301S Tau transgenic (TE4) mice with tauopathy and brain atrophy, an aberrantly higher presence of brain T cells is found to be colocalized with microglia (left panel). In the TE4 mice, depleting brain T cells by injecting anti-CD4 (αCD4) and anti-CD8 (αCD8) antibodies results in reduced tauopathy, brain atrophy, cognitive impairment, and inflammation (panel right). These findings suggest that brain T cells, including CD8^+^ T cells, exhibit a detrimental role during AD development. Interestingly, in both mouse models, PD-1 immune checkpoint blockade in TE4 mice mitigates AD pathologies (**A** & **B**). Created with BioRender (Biorender.com)

These two opposing findings raise the intriguing question: What factors contributed to the disparate conclusions regarding the role of CD8^+^ T cells in AD pathogenesis in these two studies? Here we discuss the differences between these two studies that might have contributed to the disparity. First, different AD mouse models were used. In the study conducted by Su et al., researchers utilized 5xFAD mice expressing human APP and *PSEN1* transgenes, harboring a total of five AD-linked mutations. These mice are genetically engineered for the study of the Aβ pathology. On the other hand, in the study conducted by Chen et al., researchers focused on genetically further modified P301S Tau transgenic mice that co-expressed the E4 variant of the human *APOE* (*APOE4*) gene, abbreviated as the TE4 mice [6]. P301S Tau transgenic mice carry a transgene with the P301S mutation in the tau-encoding *MAPT* gene and develop tau pathology, and co-expression of the *APOE4* gene aggravates AD-associated pathology. These two distinct models express different transgenic genes, develop different AD pathologies, and also exhibit temporal disparity in disease development [7] (see review by Yokoyama et al. [7] for more details on AD animal models and their applications). Hence, even though T cells were attracted to the brain and clonally expanded in both mouse models, the brain microenvironment for T cell proliferation and differentiation likely differed in these two studies. Secondly, the approaches employed to investigate the function of CD8^+^ T cells in the brain were different. Su et al. implemented genetic modifications in 5xFAD mice to specifically target T-cell migration and function. This included the creation of *Cxcr6*-deficient mice (blocking CXCL16-CXCR6 axis-mediated CXCR6^+^CD8^+^ T cell homing to the brain), *B2m*-deficient mice (blocking CD8^+^ T cell function), and *Tcra*-deficient mice (depleting T cells) [5] (Fig. 1A, right panel). These three approaches tackled the same question from different yet complementary angles, pinpointing CXCR6^+^ CD8^+^ T cells as the population that ameliorated AD pathologies. On the other hand, Chen et al. depleted both CD4^+^ and CD8^+^ T cells together by intraperitoneal injection of anti-CD4 and anti-CD8α antibodies (Fig. 1B, right panel), preventing brain T cell infiltration that protected mice from brain atrophy [6]. Chen et al. also reported that treatment with anti-PD-1 antibodies, previously known to ameliorate AD pathologies in both Aβ-driven and tauopathy AD mouse models [8, 9], heightened the presence of FoxP3^+^ CD4^+^ Tregs without changing the frequency of Tauopathy-associated CD8^+^ T cells [6]. Given the strong correlation between T cell brain infiltration and tauopathy, along with the presence of disease-associated microglia (DAM), Chen et al. concluded that CD8^+^ T cells infiltrating the brain were detrimental, whereas FoxP3^+^ CD4^+^ Tregs were protective in this tauopathy AD model. In this study, researchers demonstrated that the overall depletion of T cells provided protection. However, the neurodegenerative effects of tauopathy-associated CD8^+^ T cells were primarily inferred through single-cell RNA-sequencing (scRNA-seq) analysis [6]. Collectively, the utilized animal models and methodologies varied in these two studies.

Upon careful examination of these two studies, we argue that the disparate conclusions regarding CD8^+^ T cell function in neurodegeneration were likely attributable to the use of different AD mouse models. Maintaining a delicate balance among the diverse components of the immune system is essential for optimal functioning. Variations in the intricate interplay of immune elements can significantly impact the ultimate outcome of immune responses. It is well known that tumor microenvironment heavily affects CD8^+^ T cell function and differentiation [10]. Conceivably, the disparities between the AD models utilized in these two studies could collectively give rise to distinct microenvironments at diseased sites for immune cells. Research is needed to explore the effects of the brain microenvironment on CD8^+^ T cell differentiation and plasticity. Hence, the variations in the microenvironment for CD8^+^ T cells between these distinct AD mouse models [5, 6] might have led to the preferential development and expansion of specific CD8^+^ T cell subtypes, such as CD8^+^ Tregs or CTLs. It is possible that several functionally distinct subtypes of CD8^+^ T cells co-exist in a diseased brain and the collective influence on AD pathologies is driven by a dominant subtype. Su et al. illustrated in their elegant study that the clonally expanded CD8^+^ T cells around Aβ plaques functioned as regulatory cells, restraining the activation status of DAMs. However, in the tauopathy mouse model, further experimental investigation is required to validate the neurodegenerative effects attributed to tauopathy-associated CD8^+^ T cells, such as exploring tauopathy in *B2m*-deficient TE4 mice.

Regarding cell markers, Su et al. showed that the brain regulatory CD8^+^ T cells that restrained Aβ plaque deposition and cognitive decline in 5xFAD mice were CXCR6^+^PD-1^+^ [5]. PD-1 is a marker for T-cell exhaustion [11]. The finding that immune checkpoint blockade targeting PD-1 reduces AD pathologies in 5xFAD mice [9] can be used as supporting evidence to the theory that checkpoint blockade rejuvenates the protective function of exhausted PD-1^+^ regulatory CD8^+^ T cells. Interestingly, Chen et al. also observed CXCR6 and PD-1 expression in brain T cells that promoted tauopathy in TE4 mice, and reported that anti-PD-1 treatment increased the activity of PD-1^+^ CD4^+^ Tregs but did not alter the activity of detrimental PD-1^+^ CD8^+^ T cells [6]. Chen et al. implied that increased CD4^+^ Treg activity was the underlying mechanism for the previous observation that immune checkpoint blockade targeting PD-1 ameliorates tauopathy [6, 8]. It is known that both mouse and human brain CD8^+^ T cells exhibit tissue-resident memory T cell signatures, express PD-1, and are enriched for tissue-homing associated chemokine receptors, including CXCR6 [2, 12]. Hence, the phenotype of CXCR6^+^PD-1^+^ of brain T cells in both studies [5, 6] might only reflect that these were brain-resident cells instead of an association of cellular function, while the elevated PD-1 expression might merely signify the exhaustion state of these cells. It is crucial to identify the specific CD8^+^ T cell subtype(s) present in the brain and understand their rejuvenation potential when contemplating repurposing anti-PD-1 antibodies, originally designed for anticancer purposes, for the treatment of AD. Consequently, there is a need for novel markers to differentiate functionally distinct subtypes of CD8^+^ T cells in AD pathogenesis.

Clonal expansion of CD8^+^ T cells in the CNS has been linked to Alzheimer’s disease, Parkinson’s disease (PD), and multiple sclerosis (MS) in humans (reviewed by Hu et al. in [13]). We have previously argued that the similarities in cell surface markers and gene signatures between human clonally expanded CD8^+^ T cells in CSF and CD8^+^ Tregs suggest the potential existence of both protective and detrimental subsets within the neurodegeneration-associated CD8^+^ cell population [13, 14]. The two studies by Su et al. and Chen et al. in AD mouse models provide direct in vivo evidence supporting this notion. Moreover, prior to the observation of CXCL16-CXCR6 mediated CD8 T cell infiltration in the mouse models, the CXCL16-CXCR6 axis-mediated CXCR6^+^ CD8 T cell homing to cerebrospinal fluid (CSF) followed by clonal expansion of the cells has been suggested in patients with cognitive impairment [15]. These correlations between humans and mice indicate that these two studies have established valuable platforms for exploring critical questions such as which cell markers or gene signatures distinguish the protective and detrimental brain CD8^+^ T cell subtypes, how the brain microenvironment impacts the development and function of these cells, and how brain CD8^+^ T cells regulate microglia function. Considering the prevalence of clonally expanded CD8^+^ T cells in AD, PD, and MS, exploring these questions not only has the potential to reveal new insights into AD pathogenesis but also holds broader implications for the overarching field of neuroimmunology, encompassing the pathogenesis of PD and MS as well.

## Data Availability

Not applicable.

## Abbreviations

Aβ: amyloid beta
AD: Alzheimer’s disease
*APOE4*: E4 variant of the *APOE* gene
A/PE4 mouse: human *APOE4*-expressing APP/PS1-21 mouse
CD8^+^ Treg: CD8^+^ regulatory T lymphocyte
CNS: central nervous system
CTL: cytotoxic T cell
DAM: disease-associated microglia
HSC: hematopoietic stem cell
MS: multiple sclerosis
PD: Parkinson’s disease
TE4 mouse: human *APOE4*-expressing P301S Tau transgenic mouse
5xE4 mouse: human *APOE4*-expressing 5xFAD mouse
